# Frailty and hearing loss: From association to causation

**DOI:** 10.3389/fnagi.2022.953815

**Published:** 2022-09-07

**Authors:** Yun Liu, Peiyi Qian, Shuli Guo, Shuangyan Liu, Dahui Wang, Lei Yang

**Affiliations:** ^1^School of Public Health, Hangzhou Normal University, Hangzhou, China; ^2^School of Public Health, Tongji Medical College, Huazhong University of Science and Technology, Wuhan, Hubei, China

**Keywords:** Mendelian randomization, frailty index, hearing loss, causation, NAHANES

## Abstract

**Background:**

Observational studies suggest that frailty is associated with hearing loss (HL) but with inconsistent results. This study aims to examine such association and to assess its causality.

**Materials and methods:**

The cross-sectional data from the National Health and Nutrition Examination Survey (NHANES). Multivariate logistic regression models were used to assess the association between HL and frailty index (FI). Genetic variants associated with the FI and HL were obtained from a large genome-wide association study (GWAS) meta-analysis and UK Biobank GWAS. The inverse variance weighting (IVW) method was used to estimate causal effects. Sensitivity analyses were performed to further validate the robustness of results.

**Results:**

In this cross-sectional analysis, results support the possibility that frailty may be associated with a higher risk of developing HL, with self-reported [odds ratio (OR) = 2.813; 95% CI, 2.386, 3.317; *p* < 0.001], speech frequency HL (OR = 1.975; 95% CI, 1.679–2.323; *p* < 0.001), and high frequency HL (OR = 1.748; 95% CI, 1.459–2.094; *p* < 0.001). In the adjusted model, frail participants remained at high risk of HL. Mendelian randomization (MR) studies showed a bidirectional causal association between genetically predicted FI and risk of HL (FI for exposure: OR = 1.051; 95% CI, 1.020–1.083; *p* = 0.001; HL for exposure: OR = 1.527; 95% CI, 1.227–1.901; *p* < 0.001).

**Conclusion:**

Our observational study found that inter-individual differences in frailty were associated with the risk of developing HL. Genetic evidence suggests a potential bidirectional causal association between FI and HL. Furthermore, the potential mechanisms of this association require investigation.

## Introduction

Hearing loss (HL) is one of the most prevalent sensory impairments among the elderly. HL is associated with various adverse health outcomes including falls (Jiam et al., 2016; Kamil et al., 2016), depression (Lawrence et al., 2020), poor physical functioning (Chen et al., 2015), and reduced daily activities (Yamada et al., 2011; Tareque et al., 2019). According to 2019 estimates from the World Health Organization (WHO), approximately 1.57 billion people worldwide suffer from HL, a number that may increase to more than 2.45 billion by 2050 (Chadha et al., 2021). Studies on the global burden of disease have shown that HL is the third-leading cause of disability worldwide (Haile et al., 2021). Frailty is an emerging global health burden characterized by a state of non-specific vulnerability, reduced multisystem physiological reserves, and reduced resistance to stressors (Rodriguez-Manas et al., 2013). Frailty is preventable, but there is not a clear consensus on what it exactly means. Fried and his colleagues described the clinical signs of frailty using a physical phenotype. An older adult can be labeled as frail if they match three or more of the five criteria for symptoms and signs based on this frailty phenotype (Fried et al., 2001). Rockwood and Mitnitski (2007) suggested that frailty is caused by the accumulation of deficits associated with aging (Mitnitski et al., 2001). These concepts are now commonly utilized in frailty assessments. Frailty over time is associated with increased adverse health outcomes such as disability, hospitalization, and mortality (Clegg et al., 2013; Morley, 2016; Evans et al., 2022). Thus, frailty may be associated with HL or a common pathophysiological mechanism.

Evidence suggesting an association between HL and frailty and its causality is lacking, and more studies are needed to investigate the plausibility of these hypotheses. While some studies reported a positive association between HL and frailty (Doba et al., 2012; Ng et al., 2014; Buttery et al., 2015; Cakmur, 2015; Kamil et al., 2016), other studies have reported no significant correlation (Kamil et al., 2014; Liljas et al., 2017; Hamidin et al., 2018; Gu et al., 2019). As most of these studies were cross-sectional analyses limited by methodological inconsistencies, heterogeneity, and underrepresentation, Mendelian randomization (MR) analysis can address these limitations by using genetic variants that are randomly inherited at conception as unconfounded proxies for modifiable exposures to examine the potential causal effects of these exposures on disease outcomes. MR is useful for extracting causal information from observational studies and has been successfully applied to identify causal risk factors in clinical practice and to improve understanding of complex molecular signatures (Zheng et al., 2017).

We explored the correlation between HL and frailty using the National Health and Nutrition Examination Survey (NHANES) database and the established frailty index (FI) (Blodgett et al., 2015). Subsequently, their possible causal relationship was analyzed using a bidirectional two-sample MR. A causal association could allow the management of HL through frailty treatment.

## Materials and methods

### Study design and population

Data for the cross-sectional analysis were obtained from the NHANES. Participants were recruited from a random sample of residents aged 40–69 years in the years 1999–2018 (excluding 2013–2014) and were stratified by region. A total of 8,532 individuals provided the test results for audiological assessment, FI-related content, and covariate information of interest. Among these residents, 782 were excluded for missing complete hearing tests or abnormal values and 235 were excluded due to ear canal disease; thus, the analysis included 7,515 participants.

### Hearing assessment

Self-reported HL was defined as the subject selecting ‘‘moderate hearing trouble,’’ ‘‘a lot of trouble,’’ or ‘‘deaf’’ in response to the question ‘‘Which statement best describes your hearing (without a hearing aid or other listening devices)?’’ The NHANES audiometric protocol includes otoscopy, tympanometry screening, and hearing threshold tests at 0.5, 1, 2, 3, 4, 6, and 8 kHz, as described in detail elsewhere.^1^ For each participant, low- and high-frequency hearing was defined according to the WHO definition of HL as the pure tone average (PTA) calculated for each participant’s “better hearing ear” at speech frequencies (0.5, 1, 2, and 4 kHz) and high-frequency HL (3, 4, and 8 kHz) ≥20 dB HL.

### Frailty index

To assess frailty status, we used the Rockwood FI based on the cumulative model of deficits (Blodgett et al., 2015) as measured by proportion. Frailty is a continuous score of signs, and health deficits include comorbidities, psychological factors, symptoms, and disabilities (Rockwood and Mitnitski, 2007). The FI is one of the most widely used tools for measuring frailty in clinical practice and has been well-validated in the literature (Dent et al., 2016; Hoogendijk et al., 2019). For each participant, the FI value was calculated as the number of deficits present divided by the total number of 53 deficits in previous studies (all included variables are listed in Supplementary Table 1). The FI provides a continuous score from 0 (no deficits) to a theoretical maximum of 1 (all items show deficits). In addition to being a continuous variable, the FI is divided into four categories based on previously validated threshold values^24^: “non-frail,” FI ≤ 0.10; “vulnerable,” 0.10 < FI ≤ 0.21; “frail,” 0.21 < FI ≤ 0.45; and “most frail,” FI > 0.45.

### Other variables

We collected data on demographic characteristics (age, sex, race, education level, income, and marital status), physical measurements (height, weight, and blood pressure), lifestyle habits (smoking status), personal medical history [hypertension, diabetes, cardiovascular disease (CVD), and chronic obstructive pulmonary disease (COPD)], and dietary data.

### Data sources for Mendelian randomization analysis

Genetic variants significantly associated with the FI (*p* < 5 × 10^–8^) were obtained from a genome-wide association study (GWAS) meta-analysis of 164,610 UK Biobank and 10,616 TwinGene participants (Atkins et al., 2021). The FI calculations were based on 49 or 44 self-reported items on symptoms, disability, and diagnosed disorders from the UK Biobank and TwinGene databases, respectively. The GWAS meta-analysis was adjusted for age, sex, study center, and genotyping array, and the estimated single nucleotide polymorphism (SNP) heritability of the FI was 11%. The GWAS summary statistics for HL are publicly available from OpenGWAS by the UK Biobank^2^ and were used as data for the touchscreen questionnaire. The participants were assigned a case/control status based on whether they reported hearing problems (hearing difficulty/problems: yes). Among 323,978 individuals, 84,839 were cases and 239,139 were controls. All GWAS datasets used in the MR study included subjects of European ancestry. Our study was a secondary analysis of publicly available data. Informed consent was obtained from all participants according to the original GWAS protocol, and ethical approval for GWAS was obtained by the original authors.

### Statistical analyses

This study assessed the differences in sociodemographic characteristics, lifestyle, diet, and health comorbidities between hearing status categories. Wilcoxon or χ^2^ tests were used to compare the distributions of continuous or categorical variables. Logistic regression was used to examine the associations of self-reported speech frequency and high-frequency HL with frailty syndrome and to test for trends using chi-square tests. We then used multivariate logistic regression to examine the associations between different hearing states and frailty syndrome and tested all logistic regression models for the variance inflation factor (VIF). The estimates of association were expressed as dominance or odds ratio (OR) and 95% confidence interval (CI). A VIF score >5 assessed the covariance of all variables in the model. As none of the scores exceeded this threshold, all variables were retained in the model. We developed three logistic regression models: (1) adjusted for age, sex, education level, marital status, and poverty ratio; (2) additionally adjusted for body mass index (BMI), smoking status, presence of noisy work, and dietary inflammatory index; and (3) further adjusted for hypertension, diabetes, CVD, and COPD.

Bidirectional two-sample MR was used to assess the causal association between genetically predicted FI and HL. Linkage disequilibrium (LD) clumping on single-nucleotide polymorphism (SNP) data was carried out using the PLINK clumping method, among the pairs of SNPs with an LD R-square value above the specified threshold (*R*-square = 0.001) and set the window size to 10,000 kb in the resultant dataset. In addition, the alleles were coordinated based on matching to ensure independence. We identified FI-associated SNPs (Supplementary Table 2) and used them as instrumental variables in the MR analysis to test the causal association between FI and HL. Bidirectional analysis (i.e., HL as exposure and FI features as outcomes) was used to examine the direction of the association. Using the same approach, we identified genetically predicted HL-associated SNPs as instrumental variables (Supplementary Table 3).

This study used the following five methods to assess the causal effects: inverse variance weighting (IVW), MR-Egger regression, simple model, weighted model, and weighted median. The main MR analysis method was IVW regression, which is used to estimate causal effects (Hemani et al., 2018). The weighted median method was used to estimate the 50th percentile of the empirical distribution (Burgess et al., 2017) while the MR-Egger regression intercept was used to assess pleiotropy (Bowden et al., 2015). Several sensitivity analyses were performed to validate the results. The MR_Steiger method (Hemani et al., 2017) was used to test for correlations between FI and HL and to remove directionally inconsistent SNPs. The leave-one-out method was used to assess whether each SNP affected the effect of estimation. Measurement errors in SNP exposure associations were examined using the I^2^ statistic (Bowden et al., 2016). We also used Cochran’s *Q* tests to assess heterogeneity among the SNPs included in each analysis, MR-Egger regression intercept to detect horizontal multiplicity and robustness, and “MR Pleiotropy RESidual Sum and Outlier” (MR-PRESSO) (Verbanck et al., 2018) to test for multiplicity and, in obvious cases, to correct the IVW estimates by removing outliers. Our study was reported according to the Strengthening the Reporting of MR studies (STROBE-MR) guidelines (Burgess et al., 2019). All analyses were performed using the following packages in R software 4.0.3: “survey” package for considering complex survey weights in all calculations, “vcdExtra” package for the trend chi-square analysis, “var” package for covariance tests, “TwoSampleMR” package (Hemani et al., 2018) for MR analysis and the “MR_PRESSO” package (Ong and MacGregor, 2019) for multiplicity tests.

## Results

### Descriptive analyses

The weighted analysis revealed that the 7,515 NHANES participants in this study represented 38.26 million residents of the United States. Among the participants, 3910 (52.0%) were male and 3605 (48.0%) were female, with a mean (SD) age of 54.01 (8.57). Table 1 shows the characteristics of all the participants according to their hearing status.

**TABLE 1:** The characteristics of 7,515 participants with different hearing loss (HL) subtypes in the NAHANES study.

Characteristic	All subjects	Self-reported HL		Speech-frequency HL		High-frequency HL	
	*N* = 38263245.3	*N* = 10059536.6	*p*	*N* = 8622159.9	*p*	*N* = 17599311.2	*p*
Age (median [IQR])	52.0 [46.0, 60.0]	55.0 [48.0, 61.0]	<0.001	59.0 [52.0, 64.0]	<0.001	57.0 [50.0, 63.0]	<0.001
Sex = Male (%)	18250329.2 (47.7)	5841606.6 (58.1)	<0.001	5450856.6 (63.2)	<0.001	11158430.9 (63.4)	<0.001
Race (%)			<0.001		<0.001		<0.001
White	27383945.8 (71.6)	7976913.0 (79.3)		6462055.7 (74.9)		13112646.5 (74.5)	
Black	4117262.1 (10.8)	631319.4 (6.3)		635340.3 (7.4)		1412843.2 (8.0)	
Mexican American	2257876.7 (5.9)	489247.7 (4.9)		493232.6 (5.7)		1068824.3 (6.1)	
Other	4504160.8 (11.8)	962056.5 (9.6)		1031531.3 (12.0)		2004997.2 (11.4)	
Education level (%)			<0.001		<0.001		<0.001
Less than grade 9	2007927.6 (5.2)	578885.4 (5.8)		730415.0 (8.5)		1264971.7 (7.2)	
College graduate or above	12251299.6 (32.0)	2628777.7 (26.1)		1978632.1 (22.9)		4649489.3 (26.4)	
Grades 9–11 or grade 12 without diploma	3938286.3 (10.3)	1167290.8 (11.6)		1209939.2 (14.0)		2165081.6 (12.3)	
High school Graduate/GED or equivalent	8345857.5 (21.8)	2511839.9 (25.0)		2193899.9 (25.4)		4387387.3 (24.9)	
Some college or AA degree	11719874.3 (30.6)	3172742.7 (31.5)		2509273.6 (29.1)		5132381.3 (29.2)	
Smoke = yes (%)	8277368.3 (21.6)	2523541.6 (25.1)	0.009	2312164.2 (26.8)	<0.001	4580917.5 (26.0)	<0.001
Marital = Separated (%)	10822164.1 (28.3)	2937191.0 (29.2)	0.507	2582409.0 (30.0)	0.192	4984034.1 (28.3)	0.955
Poverty (median [IQR])	3.4 [2.0, 5.0]	3.2 [1.9, 5.0]	0.027	2.8 [1.7, 4.9]	<0.001	3.0 [1.8, 5.0]	<0.001
Soldier = yes (%)	4882856.1 (12.8)	1821555.9 (18.1)	<0.001	1712221.2 (19.9)	<0.001	3348127.9 (19.0)	<0.001
BMI (median [IQR])	28.4 [24.8, 32.6]	29.2 [25.6, 33.5]	<0.001	28.9 [25.4, 33.2]	<0.001	28.8 [25.4, 32.8]	<0.001
Noisework = yes (%)	11438583.1 (29.9)	4175434.0 (41.5)	<0.001	3165302.2 (36.7)	<0.001	6337936.8 (36.0)	<0.001
Dii (median [IQR])	0.8 [−0.5, 2.0]	0.9 [−0.5, 2.1]	0.94	1.0 [−0.4, 2.2]	<0.001	0.9 [−0.5, 2.0]	0.697
Hypertension = yes (%)	16911915.3 (44.2)	5149036.4 (51.2)	<0.001	4716506.6 (54.7)	<0.001	9139031.6 (51.9)	<0.001
DM = yes (%)	9256359.0 (24.2)	2999501.3 (29.8)	<0.001	2994361.2 (34.7)	<0.001	5437455.4 (30.9)	<0.001
CVD = yes (%)	3372960.1 (8.8)	1363897.3 (13.6)	<0.001	1274166.2 (14.8)	<0.001	2353149.5 (13.4)	<0.001
COPD = yes (%)	1948182.4 (5.1)	808968.5 (8.0)	<0.001	680840.7 (7.9)	<0.001	1180719.8 (6.7)	0.001

Data expressed as number (weighted% of participants) for categorical variables and median (interquartile range) of the mean for continuous variables. Wilcoxon rank-sum test was used for continuous variables. Chi-square test was used for categorical variables. All p-values are statistically significant (*p* < 0.05). HL, hearing loss; BMI, body mass index; DII, dietary inflammation index; DM, diabetes; CVD, cardiovascular disease; COPD, chronic obstructive pulmonary disease.

### Multivariable association models

The mean FI scores of the patients with self-reported, speech frequency, and high-frequency HL were 0.170 ± 0.003, 0.161 ± 0.003, and 0.152 ± 0.003, respectively. After classification into four categories, the FI scores were higher in patients with HL than in those without frailty. The results showed that frailty increased the risk of self-reported HL (OR = 2.813; 95% CI, 2.386–3.317; *p* < 0.001), speech frequency HL (OR = 1.975; 95% CI, 1.679–2.323; *p* < 0.001), and high-frequency HL (OR = 1.748; 95% CI, 1.459–2.094; *p* < 0.001), while the *p*-values for the trends were *p* < 0.001. Multivariate logistic regression analysis was adjusted for demographic and sociodemographic characteristics, lifestyle, diet, and health comorbidities. Table 2 presents the results. After full adjustment, compared to non-frail individuals, frail patients remained at a significantly higher risk of having higher HL with self-reported HL (OR = 2.780; 95% CI, 2.192–3.525; *p* < 0.001), speech frequency HL (OR = 1.549; 95% CI, 1.212–1.979; *p* < 0.001), and high-frequency HL (OR = 1.355; 95% CI, 1.058–1.736; *p* = 0.017). In addition, when stratified by gender and age, found that the risk of HL was higher in the female frail patients but not in the male; the risk of HL was increased in the frail patients aged 40–50 years and 60 years or older (Supplementary Table 4). Linear regression models indicated a linear relationship between speech- or high-frequency PTA and FI scores (Supplementary Table 5).

**TABLE 2:** Logistic regression models of frailty phenotypes as independent variables in the NAHANES study.

Variable	“Non-frail” (FI ≤ 0.10)	“Vulnerable” (0.10 < FI ≤ 0.21)	“Frail” (0.21 < FI ≤ 0.45)	“Most frail” (FI > 0.45)	*P* for trend
**Self-reported HL**					
Not adjusted	1	1.543 (1.332, 1.788)***	2.813 (2.386, 3.317)***	4.135 (2.394, 7.142)***	<0.001
Model1	1	1.650 (1.411, 1.931)***	3.127 (2.584, 3.785)***	4.514 (2.352, 8.664)***	<0.001
Model2	1	1.555 (1.342, 1.802)***	2.763 (2.264, 3.372)***	3.744 (1.964, 7.137)***	<0.001
Model3	1	1.568 (1.345, 1.829)***	2.780 (2.192, 3.525)***	3.679 (1.894, 7.147)***	<0.001
**Speech-frequency HL**					
Not adjusted	1	1.160 (0.994, 1.354)	1.975 (1.679, 2.323)***	3.300 (1.908, 5.705)***	<0.001
Model1	1	1.133 (0.947, 1.356)	1.776 (1.449, 2.176)***	2.698 (1.614, 4.512)***	<0.001
Model2	1	1.094 (0.907, 1.320)	1.632 (1.325, 2.010)***	2.199 (1.261, 3.834)**	<0.001
Model3	1	1.062 (0.862, 1.309)	1.549 (1.212, 1.979)***	2.035 (1.134, 3.652)*	<0.001
**High-frequency HL**					
Not adjusted	1	1.059 (0.927, 1.210)	1.748 (1.459, 2.094)***	2.419 (1.243, 4.706)*	<0.001
Model1	1	1.071 (0.904, 1.270)	1.725 (1.396, 2.131)***	2.127 (1.039, 4.354)*	<0.001
Model2	1	1.031 (0.869, 1.222)	1.544 (1.246, 1.912)***	1.616 (0.796, 3.281)	<0.001
Model3	1	0.979 (0.821, 1.167)	1.355 (1.058, 1.736)*	1.248 (0.607, 2.569)	0.020

Data expressed as odds ratio (95% confidence interval). Model1 = Multivariate logistic regression model adjusted for age, gender, race, education, and poverty ratio, marital, and military. Model2 = model1 addition smoking status, body mass index, noisework, and dietary inflammation index. Model3 = mode2 addition hypertension, diabetes, cardiovascular disease, and chronic obstructive pulmonary disease. FI, frailty index. Speech-frequency HL: Defined as profound hearing impairment at frequencies of 500, 1000, 2000, and 4000 Hz. High-frequency HL: Defined as profound hearing impairment at frequencies of 3000, 4000, and 8000 Hz. Significance codes: “***” <0.001, “**” <0.01, “*” <0.05.

### Mendelian randomization analysis

**FIGURE 1:**
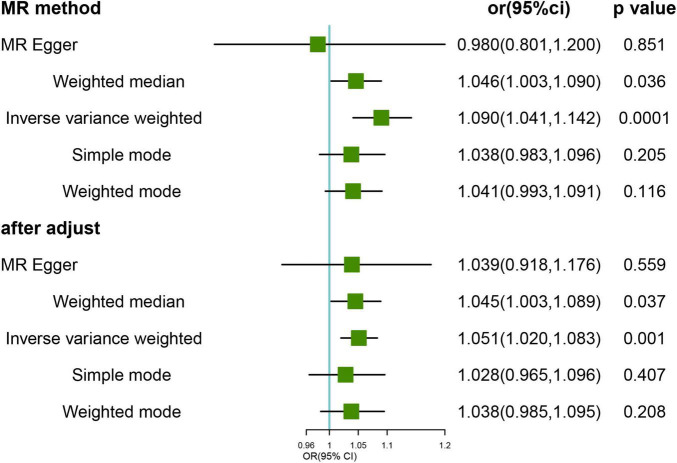
Mendelian randomization (MR) results for genetically predicted frailty index (FI) and hearing loss (HL). OR, odds ratio; CI, confidence interval. After adjust: after removing the three outlier SNPs (rs1363103, rs17612102, and rs82334).

Mendelian randomization analysis of genetic variants associated with FI showed a potential causal association between genetically predicted FI and the risk of developing HL. The main IVW analysis method (OR = 1.090; 95% CI, 1.041–1.141; *p* < 0.001) (Figure 1) showed corrected results after removing the three outlier SNPs (rs1363103, rs17612102, and rs82334) using the MR-PRESSO test, indicating a persisting causal association (OR = 1.051, 95% CI, 1.020–1.083; *p* = 0.001). Sensitivity analysis showed no heterogeneity (all *p* > 0.05) or pleiotropy (all *p* > 0.05) in the estimated effect of FI on HL after the removal of outliers (Supplementary Table 6). Leave-one-out analysis showed that the estimated effects were relatively stable when a single SNP was excluded (Supplementary Figure 1).

**FIGURE 2:**
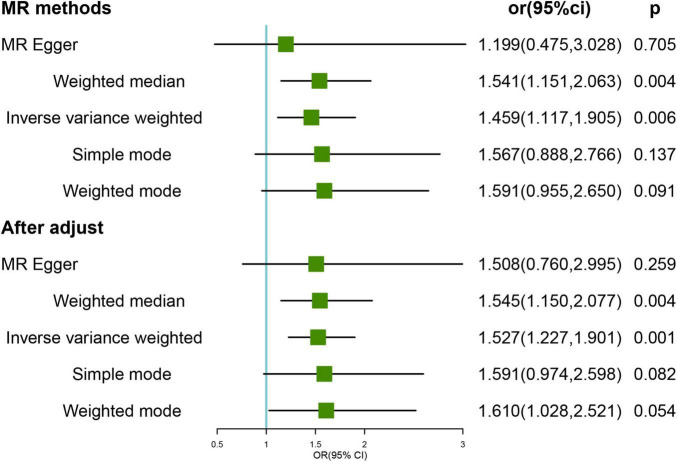
Mendelian randomization (MR) results for genetically predicted hearing loss (HL) and frailty index (FI). OR, odds ratio; CI, confidence interval. After adjust: after removing the four outlier SNPs (rs34656207, rs741475, rs10901863, rs1126809, rs1566129, and rs9296413).

We tested the direction of the association using genetically predicted HL as the exposure and FI as the outcome. The results of the primary IVW analysis showed a potential causal association between genetically predicted HL and the risk of frailty (OR = 1.459, 95% CI, 1.117–1.905; *p* = 0.006) (Figure 2). The outlier-corrected IVW estimates were not significantly altered (OR = 1.527; 95% CI, 1.227–1.901; *p* < 0.001) by removing the four outlier SNPs (rs10901863, rs1126809, rs1566129, and rs9296413) according to the MR-PRESSO tests. Sensitivity analysis showed no heterogeneity (all *p* > 0.05) or pleiotropy (all *p* > 0.05) in the estimated effect of HL on FI after the removal of outliers (Supplementary Table 5). Leave-one-out analysis showed that the estimated effects were relatively stable when single SNPs were excluded (Supplementary Figure 2).

## Discussion

The cross-sectional analysis in the current study identified an association between frailty and hearing; the correlation between FI and HL remained significant after adjusting for potential confounders of demographic factors, lifestyle, dietary factors, and chronic disease. In addition, a bidirectional two-sample MR analysis revealed a bidirectional causal relationship between FI and HL. To our knowledge, this is the first study to examine the association between HL and FI using MR.

Frailty is a multidimensional syndrome that spans the sociodemographic, clinical, lifestyle-related, and biological domains. Most available research on HL focuses on individual-level goals, whereas few studies have focused on system-level goals. Notably, relatively few conflicting studies on HL and FI have been conducted. In a meta-analysis of hearing and frailty, pooled data from 10 cross-sectional studies showed a 61% increased risk of developing HL in pre-frail individuals compared to non-frail individuals (OR = 1.61, 95% CI = 1.28–2.01, *N* = 14,329), while HL was associated with a 2.5-fold increased odds of frailty (Tan et al., 2020) (OR = 2.53, 95% CI = 1.88–3.41, *N* = 9,322). A recent meta-analysis of 12 cross-sectional (*N* = 12,313) and three longitudinal (*N* = 3042) studies showed similar findings, with HL associated with 87% (OR = 1.87, 95% CI = 1.63–2.13) and 56% (OR = 1.56, 95% CI = 1.29–1.88) increased risks of frailty. However, several cross-sectional (Ng et al., 2014; Gu et al., 2019; Lorenzo-Lopez et al., 2019) and longitudinal studies (Liljas et al., 2017; Trevisan et al., 2017) reported no association between frailty and HL. Most of these studies used self-reports and simple measures of hearing (e.g., whisper or finger rub tests), with no standard definition of frailty, and also showed a high degree of heterogeneity. A recent study from the NHANES showed increased odds of low-frequency HL in frail individuals compared to non-frail individuals based on measured HL (OR = 4.01, 95% CI = 1.27–12.63), with no association for high-frequency HL. PTA was linearly associated with FI (low: β = 10.15; 95% CI, 1.78–18.51; high: β = 19.85; 95% CI, 5.19–34.53) (Hura et al., 2021) and FI and HL occurrence may be dose-dependent, similar to our results obtained by using the WHO-defined PTA frequencies to assess HL and decibel cut-off values. In the present study, a relatively large sample size and pure tone measures of HL showed an increased risk of HL occurrence in frail patients compared to non-frail individuals (including self-reported, speech frequency, and high-frequency HL) after adjusting for confounding factors. Our results also revealed a possible bidirectional causal association between FI and HL using a bidirectional two-sample MR analysis, which avoided confounding factors and reverse causality.

Previous evidence showed that frailty is associated with HL and aging, and may also be related to shared age-related degenerative changes. Underlying cellular aging is also associated with environmental, genetic, and chronic disease states that promote both HL (Lin et al., 2016; Wattamwar et al., 2018) and the development of a frailty phenotype (Hingorani et al., 2013; Walston et al., 2019). The reported neurobiological factors associated with age-related hearing loss (ARHL) and physical decline include inflammatory processes and primitive neurodegeneration of the auditory cortex as well as nutritional, vascular, neuropathological, and metabolic factors (Robertson et al., 2013; Ruan et al., 2015). Recent studies have found that age-related central auditory processing disorder (CAPD) is independently associated with cognitive frailty but not physical frailty, suggesting that CAPD rather than peripheral components may play a role in accelerated cognitive performance frailty associated with aging (Sardone et al., 2021). This result is in line with findings from previous population-based research (Panza et al., 2015). Investigators suggest that the association between age-related CAPD and cognition may be related to age-related degeneration in specific brain regions and that age-related CAPD is not associated with physical frailty may be attributable to mechanisms of a frailty phenotype determined primarily by the aging process. Hearing difficulties can impede appropriate physical responses, and comorbidities may also indicate the impairment of potentially common mechanisms, such as the regulation of responses to stress, impaired immunity, and inflammatory responses (Spankovich and Le Prell, 2013). In addition, several factors associated with physical frailty may be related to cognitive impairment, including inflammatory markers and vascular factors, which may also directly contribute to ARHL. GWAS studies have found that most FI-related loci are associated with features such as BMI, CVD, smoking, human leukocyte antigen (HLA) proteins, depression, and neuroticism and contain many HLA genes (Atkins et al., 2021). HLA is a cell surface protein essential for the regulation of immune function and is known to decline with age. Deafness has also been associated with HLA genotypes in two families (Deschamps et al., 1983). Although many associations between hearing and FI may occur due to a common underlying pathological process, the biological mechanisms behind its development are still far from being understood. Given that frailty is dynamic and preventable, up to a likely point of no return when it enters a pre-death phase (Puts et al., 2017). Thence, exploring its association with HL in detail is crucial. Future studies with comprehensive experiments, animal models, and randomized controlled trials on the prevention or treatment of frailty are needed to better understand these mechanisms.

This study had several strengths and weaknesses. Among the strength were the use of self-reports and PTA to assess hearing, in addition to the well-represented and accurate study population. To more accurately assess the development of frailty in the cumulative model of deficits, we expanded the content of the FI components to include more comorbidities, cognitive functioning, and social isolation in the analysis. This study was the first to use MR to assess the bidirectional causal association between FI and HL, thereby avoiding possible confounders and reverse causation.

The limitations of this study were as follows. First, population stratification was a potential source of bias in the MR analysis. Although Atkins et al. (2021) adjusted for age and sex stratification in their GWAS analysis, populations with low education and high BMI had a higher risk of frailty, which may lead to bias. Second, frailty and HL may be polygenic. Although the current study used the MR-Egger intercept and MR_PRESSO tests, the results showed no pleiotropy. Future studies could also use the Causal Analysis Using Summary Effect Estimates (CAUSE) analysis and multivariate MR to verify causality. Third the current MR study was limited to participants of European ancestry; thus, the results may not be generalizable to other study populations. Fourth, we note the existence of different definitions of vulnerability, and the use of different definitions may affect the results of the analysis. Finally, we were unable to identify the mechanistic pathways by which HL is associated with frailty. Although GWAS has had great success in studying genetic susceptibility to complex diseases and traits, the biological mechanisms underlying most of the genetic variants it has identified remain unclear, which will pose a challenge to causal inference in MR studies.

## Conclusion

Our results in a nationally representative sample of population-based individuals in the United States support the possibility that frailty is associated with a higher risk of developing HL. Specifically, self-reported, speech frequency, and high frequency HL were independently associated with FI. The MR analysis identified a possible bidirectional causal association between FI and HL. Further studies are needed to reveal potential common biological pathways or identify modifiable debilitating factors in HL to suggest personalized prevention and treatment.

## Data availability statement

The original contributions presented in this study are included in the article/Supplementary material, further inquiries can be directed to the corresponding authors.

## Ethics statement

Ethical review and approval was not required for the study on human participants in accordance with the local legislation and institutional requirements. Written informed consent to participate in this study was provided by the participants or their legal guardian/next of kin.

## Author contributions

YL, DW, and LY conducted the study design and revised the manuscript. YL oversaw data analysis and wrote the manuscript. SL edited the manuscript. SG and PQ conducted the statistical analysis. DW and LY critically revised the final manuscript. All authors contributed to the article and approved the submitted version.

## Footnotes

1
https://www.cdc.gov/nchs/nhanes/index.htm

2
https://gwas.mrcieu.ac.uk/

## Supplementary material

Supplementary Figure 1 Sensitivity analysis of the association between genetically predicted frailty index (FI) and risk of hearing loss (HL). **(A)** Leave-one-out analysis of the effect of genetically predicted FI and HL; **(B)** forest plot of the effect of genetically predicted FI and HL; **(C)** scatter plot of the effect of genetically predicted FI and HL; **(D)** funnel plot of the effect of genetically predicted FI and HL.

Supplementary Figure 2 Sensitivity analysis of the association between genetically predicted hearing loss (HL) and risk of frailty index (FI). **(A)** Leave-one-out analysis of the effect of genetically predicted HL and FI; **(B)** forest plot of the effect of genetically predicted HL and FI; **(C)** scatter plot of the effect of genetically predicted FI and HL; **(D)** funnel plot of the effect of genetically predicted FI and HL.

## References

[B1] Atkins J. L. Jylhava J. Pedersen N. L. Magnusson P. K. Lu Y. Wang Y. (2021). A genome-wide association study of the frailty index highlights brain pathways in ageing. *Aging Cell.* 20:e13459. 10.1111/acel.13459 34431594 PMC8441299

[B2] Blodgett J. Theou O. Kirkland S. Andreou P. Rockwood K. (2015). Frailty in NHANES: Comparing the frailty index and phenotype. *Arch. Gerontol. Geriatr.* 60 464–470. 10.1016/j.archger.2015.01.016 25697060

[B3] Bowden J. Del Greco M. F. Minelli C. Davey Smith G. Sheehan N. A. Thompson J. R. (2016). Assessing the suitability of summary data for two-sample Mendelian randomization analyses using MR-Egger regression: The role of the I2 statistic. *Int. J. Epidemiol.* 45 1961–1974. 10.1093/ije/dyw220 27616674 PMC5446088

[B4] Bowden J. Smith G. D. Burgess S. (2015). Mendelian randomization with invalid instruments: Effect estimation and bias detection through Egger regression. *Int. J. Epidemiol.* 44 512–525. 10.1093/ije/dyv080 26050253 PMC4469799

[B5] Burgess S. Bowden J. Fall T. Ingelsson E. Thompson S. G. (2017). Sensitivity Analyses for Robust Causal Inference from Mendelian Randomization Analyses with Multiple Genetic Variants. *Epidemiology* 28 30–42. 10.1097/EDE.0000000000000559 27749700 PMC5133381

[B6] Burgess S. Davey Smith G. Davies N. M. Dudbridge F. Gill D. Glymour M. M. (2019). Guidelines for performing Mendelian randomization investigations. *Wellcome Open Res.* 4:186. 10.12688/wellcomeopenres.15555.2 32760811 PMC7384151

[B7] Buttery A. K. Busch M. A. Gaertner B. Scheidt-Nave C. Fuchs J. (2015). Prevalence and correlates of frailty among older adults: Findings from the German health interview and examination survey. *BMC Geriatr.* 15:22. 10.1186/s12877-015-0022-3 25879568 PMC4357063

[B8] Cakmur H. (2015). Frailty Among Elderly Adults in a Rural Area of Turkey. *Med. Sci. Monitor* 21 1232–1242. 10.12659/Msm.893400 25925800 PMC4427024

[B9] Chadha S. Kamenov K. Cieza A. (2021). The world report on hearing, 2021. *Bull. World Health Organiz.* 99:242. 10.2471/Blt.21.285643 33953438 PMC8085630

[B10] Chen D. S. Betz J. Yaffe K. Ayonayon H. N. Kritchevsky S. Martin K. R. (2015). Association of hearing impairment with declines in physical functioning and the risk of disability in older adults. *J. Gerontol. A Biol. Sci. Med. Sci.* 70 654–661. 10.1093/gerona/glu207 25477427 PMC4400396

[B11] Clegg A. Young J. Iliffe S. Rikkert M. O. Rockwood K. (2013). Frailty in elderly people. *Lancet* 381 752–762. 10.1016/S0140-6736(12)62167-9 23395245 PMC4098658

[B12] Dent E. Kowal P. Hoogendijk E. O. (2016). Frailty measurement in research and clinical practice: A review. *Europ. J. Int. Med.* 31 3–10. 10.1016/j.ejim.2016.03.007 27039014

[B13] Deschamps I. Lestradet H. Schmid M. Hors J. (1983). HLA-DR2 and DIDMOAD syndrome. *Lancet* 2:109. 10.1016/s0140-6736(83)90096-x 6134946

[B14] Doba N. Tokuda Y. Goldstein N. E. Kushiro T. Hinohara S. (2012). A pilot trial to predict frailty syndrome: The Japanese Health Research Volunteer Study. *Exp. Gerontol.* 47 638–643. 10.1016/j.exger.2012.05.016 22664579

[B15] Evans N. R. Todd O. M. Minhas J. S. Fearon P. Harston G. W. Mant J. (2022). Frailty and cerebrovascular disease: Concepts and clinical implications for stroke medicine. *Int. J. Stroke* 17 251–259. 10.1177/17474930211034331 34282986 PMC8864332

[B16] Fried L. P. Tangen C. M. Walston J. Newman A. B. Hirsch C. Gottdiener J. (2001). Frailty in older adults: Evidence for a phenotype. *J. Gerontol. A Biol. Sci. Med. Sci.* 56 M146–M156. 10.1093/gerona/56.3.m146 11253156

[B17] Gu J. Chen H. Y. Gu X. Q. Sun X. M. Pan Z. G. Zhu S. Z. (2019). Frailty and associated risk factors in elderly people with health examination in rural areas of China. *Iran. J. Publ. Health* 48 1663–1670. PMC6825679 31700822

[B18] Haile L. M. Kamenov K. Briant P. S. Orji A. U. Steinmetz J. D. Abdoli A. (2021). Hearing loss prevalence and years lived with disability, 1990-2019: Findings from the Global Burden of Disease Study 2019. *Lancet* 397 996–1009. 33714390 10.1016/S0140-6736(21)00516-X PMC7960691

[B19] Hamidin F. A. M. Adznam S. N. Ibrahim Z. Chan Y. M. Aziz N. H. A. (2018). Prevalence of frailty syndrome and its associated factors among community-dwelling elderly in East Coast of Peninsular Malaysia. *Sage Open Med.* 6:2050312118775581. 10.1177/2050312118775581 29872529 PMC5977425

[B20] Hemani G. Tilling K. Davey Smith G. (2017). Orienting the causal relationship between imprecisely measured traits using GWAS summary data. *PLoS Genet* 13:e1007081. 10.1371/journal.pgen.1007081 29149188 PMC5711033

[B21] Hemani G. Zheng J. Elsworth B. Wade K. H. Haberland V. Baird D. (2018). The MR-Base platform supports systematic causal inference across the human phenome. *Elife* 7:e34408. 10.7554/eLife.34408 29846171 PMC5976434

[B22] Hingorani A. D. van der Windt D. A. Riley R. D. Abrams K. Moons K. G. M. Steyerberg E. W. (2013). Prognosis research strategy (PROGRESS) 4: Stratified medicine research. *BMJ* 346:e5793. 10.1136/bmj.e5793 23386361 PMC3565686

[B23] Hoogendijk E. O. Afilalo J. Ensrud K. E. Kowal P. Onder G. Fried L. P. (2019). Frailty: Implications for clinical practice and public health. *Lancet* 394 1365–1375. 10.1016/S0140-6736(19)31786-6 31609228

[B24] Hura N. Bernstein I. A. Mady L. J. Agrawal Y. Lane A. P. Rowan N. R. (2021). Otolaryngic sensory loss as a measure of frailty among older US adults. *Int. Forum Allerg. Rhinol.* 12 771–779. 10.1002/alr.22918 34878232

[B25] Jiam N. T. Li C. Agrawal Y. (2016). Hearing loss and falls: A systematic review and meta-analysis. *Laryngoscope* 126 2587–2596. 10.1002/lary.25927 27010669

[B26] Kamil R. J. Betz J. Powers B. B. Pratt S. Kritchevsky S. Ayonayon H. N. (2016). Association of hearing impairment with incident frailty and falls in older adults. *J. Aging Health* 28 644–660. 10.1177/0898264315608730 26438083 PMC5644033

[B27] Kamil R. J. Li L. S. Lin F. R. (2014). Association between hearing impairment and frailty in older adults. *J. Am. Geriatr. Soc.* 62 1186–1188. 10.1111/jgs.12860 24925554 PMC4141776

[B28] Lawrence B. J. Jayakody D. M. P. Bennett R. J. Eikelboom R. H. Gasson N. Friedland P. L. (2020). Hearing loss and depression in older adults: A systematic review and meta-analysis. *Gerontologist* 60 e137–e154. 10.1093/geront/gnz009 30835787

[B29] Liljas A. E. M. Carvalho L. A. Papachristou E. Oliveira C. Wannamethee S. G. Ramsay S. E. (2017). Self-reported hearing impairment and incident frailty in english community-dwelling older adults: A 4-Year Follow-Up Study. *J. Am. Geriatr. Soc.* 65 958–965. 10.1111/jgs.14687 27991672 PMC5484326

[B30] Lin B. M. Curhan S. G. Wang M. Eavey R. Stankovic K. M. Curhan G. C. (2016). Hypertension, diuretic use, and risk of hearing loss. *Am. J. Med.* 129 416–422. 10.1016/j.amjmed.2015.11.014 26656761 PMC4792671

[B31] Lorenzo-Lopez L. Lopez-Lopez R. Maseda A. Bujan A. Rodriguez-Villamil J. L. Millan-Calenti J. C. (2019). Changes in frailty status in a community-dwelling cohort of older adults: The VERISAUDE study. *Maturitas* 119 54–60. 10.1016/j.maturitas.2018.11.006 30502751

[B32] Mitnitski A. B. Mogilner A. J. Rockwood K. (2001). Accumulation of deficits as a proxy measure of aging. *Scientif. World J.* 1 323–336. 10.1100/tsw.2001.58 12806071 PMC6084020

[B33] Morley J. E. (2016). Frailty and Sarcopenia: The New Geriatric Giants. *Rev. Investig. Clin. Clin. Transl. Investig.* 68 59–67. 27103041

[B34] Ng T. P. Feng L. Nyunt M. S. Larbi A. Yap K. B. (2014). Frailty in older persons: Multisystem risk factors and the Frailty Risk Index (FRI). *J. Am. Med. Dir. Assoc.* 15 635–642. 10.1016/j.jamda.2014.03.008 24746590

[B35] Ong J. S. MacGregor S. (2019). Implementing MR-PRESSO and GCTA-GSMR for pleiotropy assessment in Mendelian randomization studies from a practitioner’s perspective. *Genet. Epidemiol.* 43 609–616. 10.1002/gepi.22207 31045282 PMC6767464

[B36] Panza F. Solfrizzi V. Logroscino G. (2015). Age-related hearing impairment-a risk factor and frailty marker for dementia and AD. *Nat. Rev. Neurol.* 11 166–175. 10.1038/nrneurol.2015.12 25686757

[B37] Puts M. T. E. Toubasi S. Andrew M. K. Ashe M. C. Ploeg J. Atkinson E. (2017). Interventions to prevent or reduce the level of frailty in community-dwelling older adults: A scoping review of the literature and international policies. *Age Ageing* 46 383–392. 10.1093/ageing/afw247 28064173 PMC5405756

[B38] Robertson D. A. Savva G. M. Kenny R. A. (2013). Frailty and cognitive impairment–a review of the evidence and causal mechanisms. *Ageing Res. Rev.* 12 840–851. 10.1016/j.arr.2013.06.004 23831959

[B39] Rockwood K. Mitnitski A. (2007). Frailty in relation to the accumulation of deficits. *J. Gerontol. A Biol. Sci. Med. Sci.* 62 722–727. 10.1093/gerona/62.7.722 17634318

[B40] Rodriguez-Manas L. Feart C. Mann G. Vina J. Chatterji S. Chodzko-Zajko W. (2013). Searching for an operational definition of frailty: A Delphi method based consensus statement: The frailty operative definition-consensus conference project. *J. Gerontol. A Biol. Sci. Med. Sci.* 68 62–67. 10.1093/gerona/gls119 22511289 PMC3598366

[B41] Ruan Q. Yu Z. Chen M. Bao Z. Li J. He W. (2015). Cognitive frailty, a novel target for the prevention of elderly dependency. *Ageing Res. Rev.* 20 1–10. 10.1016/j.arr.2014.12.004 25555677

[B42] Sardone R. Castellana F. Bortone I. Lampignano L. Zupo R. Lozupone M. (2021). Association between central and peripheral age-related hearing loss and different frailty phenotypes in an older population in Southern Italy. *JAMA Otolaryngol. Head Neck Surg.* 147 561–571. 10.1001/jamaoto.2020.5334 33570584 PMC7879383

[B43] Spankovich C. Le Prell C. G. (2013). Healthy diets, healthy hearing: National Health and Nutrition Examination Survey, 1999-2002. *Int. J. Audiol.* 52 369–376. 10.3109/14992027.2013.780133 23594420 PMC4036465

[B44] Tan B. K. J. Man R. E. K. Gan A. T. L. Fenwick E. K. Varadaraj V. Swenor B. K. (2020). Is sensory loss an understudied risk factor for frailty? A systematic review and meta-analysis. *J. Gerontol. A Biol. Sci. Med. Sci.* 75 2461–2470. 10.1093/gerona/glaa171 32735331

[B45] Tareque M. I. Chan A. Saito Y. Ma S. Malhotra R. (2019). the impact of self-reported vision and hearing impairment on health expectancy. *J. Am. Geriatr. Soc.* 67 2528–2536. 10.1111/jgs.16086 31411348

[B46] Trevisan C. Veronese N. Maggi S. Baggio G. Toffanello E. D. Zambon S. (2017). Factors influencing transitions between frailty states in elderly adults: The progetto veneto anziani longitudinal study. *J. Am. Geriatr. Soc.* 65 179–184. 10.1111/jgs.14515 27861714

[B47] Verbanck M. Chen C. Y. Neale B. Do R. (2018). Detection of widespread horizontal pleiotropy in causal relationships inferred from Mendelian randomization between complex traits and diseases (vol 50, 693, 2018). *Nat. Genet.* 50 1196–1196. 10.1038/s41588-018-0164-2 29686387 PMC6083837

[B48] Walston J. Bandeen-Roche K. Buta B. Bergman H. Gill T. M. Morley J. E. (2019). Moving frailty toward clinical practice: NIA intramural frailty science symposium summary. *J. Am. Geriatr. Soc.* 67 1559–1564. 10.1111/jgs.15928 31045254 PMC6830521

[B49] Wattamwar K. Qian Z. J. Otter J. Leskowitz M. J. Caruana F. F. Siedlecki B. (2018). Association of cardiovascular comorbidities with hearing loss in the older old. *JAMA Otolaryngol.-Head Neck Surg.* 144 623–629. 10.1001/jamaoto.2018.0643 29902313 PMC6145783

[B50] Yamada M. Nishiwaki Y. Michikawa T. Takebayashi T. (2011). Impact of hearing difficulty on dependence in activities of daily living (ADL) and mortality: A 3-year cohort study of community-dwelling Japanese older adults. *Arch. Gerontol. Geriatr.* 52 245–249. 10.1016/j.archger.2010.04.023 20546947

[B51] Zheng J. Baird D. Borges M. C. Bowden J. Hemani G. Haycock P. (2017). Recent developments in mendelian randomization studies. *Curr. Epidemiol. Rep.* 4 330–345. 10.1007/s40471-017-0128-6 29226067 PMC5711966

