# Supplementary material for: Frailty and hearing loss: From association to causation
Source: Front Aging Neurosci. 2022 Sep 7;14:953815. doi: 10.3389/fnagi.2022.953815 (PMC9490320; doi:10.3389/fnagi.2022.953815)
Supplement: Supplementary file 1 [file Table_1.DOCX]

**Supplementary table 1. The 53-Item Frailty Index and Their Respective Scorings**

| **Item** | **Scoring** |
| --- | --- |
| **Cognition** |  |
| 1. experience confusion/memory problems | yes=1; no=0 |
| **Dependence** |  |
| 2. managing money difficulty | no difficulty=0; some difficulty=0.33; much difficulty=0.66; unable to do=1 |
| 3. walking for a quarter mile difficulty | the same to above |
| 4. walking up ten steps difficulty | the same to above |
| 5. stooping, crouching, kneeling difficulty | the same to above |
| 6. lifting or carrying difficulty | the same to above |
| 7. house chore difficulty | the same to above |
| 8. preparing meals difficulty | the same to above |
| 9. walking between rooms on same floor | the same to above |
| 10. standingup from armless chair difficulty | the same to above |
| 11. getting in and out of bed difficulty | the same to above |
| 12. using fork, knife, drinking from cup difficulty | the same to above |
| 13. dressing yourself difficulty | the same to above |
| 14. standing for long periods difficulty | the same to above |
| 15. Sitting for long periods difficulty | the same to above |
| 16. reaching up over head difficulty | the same to above |
| 17. grasp/holding small objects difficulty | the same to above |
| 18. going out to movies, events difficulty | the same to above |
| 19. attending social event difficulty | the same to above |
| 20. leisure activity at home difficulty | the same to above |
| 21. push or pull large objects difficulty | the same to above |
| **Depressive Symptoms** |  |
| 22. have little interest in doing things | ~2003 every day, nearly every day = 1 most days = 0.75 about half the days = 0.50 less than half the days = 0.25 2005~ nearly every day = 1 more than half the days = 0.66 several days = 0.33 |
| 23. feeling down, depressed, or hopeless | the same to above |
| 24. trouble sleeping or sleeping too much | ~2003 every night = 1 nearly every night = 0.66 less often = 0.33 2005~ nearly every day = 1 more than half the days = 0.66 several days = 0.33 |
| 25. feeling tired or having little energy | nearly every day = 1 more than half the days = 0.66 several days = 0.33 |
| 26. poor appetite or overeating | ~2003 yes = 1 no = 0 2005~ the same to above |
| 27. feeling bad about yourself | the same to above |
| 28. trouble concentrating on things | the same to above |
| **Comorbidities** |  |
| 29. doctor ever said you had arthritis | yes = 1; no = 0 |
| 30. ever told you had thyroid problem | the same to above |
| 31. ever told you had chronic bronchitis | the same to above |
| 32. ever told you had cancer or malignancy | the same to above |
| 33. ever told had congestive heart failure | the same to above |
| 34. ever told you had coronary heart disease | the same to above |
| 35. ever told you had angina/angina pectoris | the same to above |
| 36. ever told you had heart attack | the same to above |
| 37. ever told you had a stroke | the same to above |
| 38. ever told you had high blood pressure | the same to above |
| 39. doctor told you have diabetes | yes = 1; no =0; borderline=0.5 |
| 40. ever told you had weak/failing kidneys | yes = 1; no =0 |
| 41. urine leakage bother you? | 1999 yes = 1 ; no = 0 2001~ greatly = 1 very much = 0.75 somewhat = 0.5 only a little = 0.25 |
| **Hospital Utilization and Access to Care** |  |
| 42. general health condition | excellent, very good, good = 0 fair, poor = 1 |
| 43. health now compared with 1 year ago | about the same, better = 0 worse = 1 |
| 44. overnight hospital patient in last year | yes = 1, no = 0 |
| 45. times receive healthcare over past year | none = 0; 1-4 = 0.5; >=5 =1 |
| 46. number of prescription medicines taken | no = 0; 1-4 = 0.5; >=5 =1 |
| **Physical Performance and Anthropometry** |  |
| 47. body mass index (kg/m^2) | <18.5, ≥30 = 1 25–<30 = 0.5 18.5–25 = 0 |
| **Laboratory Values** |  |
| 48. glycohemoglobin(%) | 0%–5.7% = 0, >5.7% = 1 |
| 49. red blood cell count (million cells/ul) | M: 4.7–6.1 = 0, Other = 1 F: 4.2–5.4 = 0, Other = 1 |
| 50. hemoglobin (g/dl) | M: 13.5–18 = 0, Other = 1 F: 12–16 = 0, Other = 1 |
| 51. red cell distribution width (%) | 11.6–14.6 = 0, Other = 1 |
| 52. lymphocyte percent (%) | 20–40 = 0, Other = 1 |
| 53. segmented neutrophils percent (%) | 40–80 = 0, Other = 1 |
