# Supplementary material for: Frailty and hearing loss: From association to causation
Source: Front Aging Neurosci. 2022 Sep 7;14:953815. doi: 10.3389/fnagi.2022.953815 (PMC9490320; doi:10.3389/fnagi.2022.953815)
Supplement: Supplementary file 2 [file Table_2.DOCX]

| **SNP** | **Nearby gene** | **effect_allele** | **other_allele** | **EAF** | **Beta** | **SE** | **P-Value** | **F statistic** |
| --- | --- | --- | --- | --- | --- | --- | --- | --- |
| rs12739243 | *SYT14* | T | C | 0.779 | 0.0242 | 0.004 | 1.28E-09 | 34.6 |
| rs4952693 | *LRPPRC* | T | C | 0.373 | -0.0194 | 0.0034 | 1.47E-08 | 29.5 |
| rs2071207 | *SEMA3F-AS1* | T | C | 0.522 | 0.0187 | 0.0033 | 1.47E-08 | 31.6 |
| rs583514 | *NLGN1* | T | C | 0.489 | -0.0199 | 0.0033 | 1.65E-09 | 35 |
| rs82334 | *HTT* | A | C | 0.682 | 0.0223 | 0.0035 | 3.13E-10 | 36.9 |
| rs1363103 | *–* | T | C | 0.620 | 0.0191 | 0.0034 | 2.23E-08 | 29.8 |
| rs9275160 | *HLA-DQB1* | A | G | 0.340 | 0.0382 | 0.0035 | 7.18E-28 | 113.6 |
| rs2396766 | *FOXP2* | A | G | 0.473 | 0.0201 | 0.0033 | 1.22E-09 | 34.9 |
| rs56299474 | *REEP4* | A | C | 0.173 | 0.0241 | 0.0044 | 3.94E-08 | 28.5 |
| rs4146140 | *ANK3* | T | C | 0.381 | -0.0198 | 0.0034 | 6.83E-09 | 33 |
| rs10891490 | *NCAM1* | T | C | 0.409 | 0.0188 | 0.0034 | 2.00E-08 | 30.6 |
| rs3959554 | *EXD1, INO80* | A | G | 0.582 | -0.0189 | 0.0034 | 1.74E-08 | 30.8 |
| rs17612102 | *LEO1, MAPK6* | T | C | 0.407 | -0.0187 | 0.0034 | 2.85E-08 | 30.6 |
| rs8089807 | *KC6, PIK3C3* | T | C | 0.187 | -0.0248 | 0.0043 | 6.50E-09 | 33.7 |

**Supplementary Table 2. Characteristics of SNPs predictive of frailty index (FI).**

SNP: single nucleotide polymorphism; MAF: minor allele frequency; SE: standard error.
