# Supplementary material for: Frailty and hearing loss: From association to causation
Source: Front Aging Neurosci. 2022 Sep 7;14:953815. doi: 10.3389/fnagi.2022.953815 (PMC9490320; doi:10.3389/fnagi.2022.953815)
Supplement: Supplementary file 3 [file Table_3.DOCX]

**Supplementary Table 3. Characteristics of SNPs predictive of hearing loss (HL).**

| **SNP** | **Nearby gene** | **effect_allele** | **other_allele** | **EAF** | **Beta** | **SE** | **P-Value** | **F statistic** |
| --- | --- | --- | --- | --- | --- | --- | --- | --- |
| rs7525101 | *LMX1A* | T | C | 0.440 | 0.006 | 0.001 | 3.65E-09 | 72.884 |
| rs741475 | *AC007879.2* | T | C | 0.578 | -0.006 | 0.001 | 4.09E-09 | 70.952 |
| rs4859223 | *TMEM207* | A | T | 0.331 | -0.007 | 0.001 | 3.08E-10 | 69.267 |
| rs13147559 | *CLRN2* | G | C | 0.133 | 0.009 | 0.002 | 4.36E-08 | 31.558 |
| rs13172686 | *ARHGEF28* | C | T | 0.471 | 0.010 | 0.001 | 1.25E-19 | 112.236 |
| rs9493627 | *EYA4* | A | G | 0.320 | 0.008 | 0.001 | 1.95E-11 | 71.535 |
| rs34656207 | *TBC1D22B* | T | C | 0.368 | 0.007 | 0.001 | 4.75E-09 | 69.392 |
| rs6902016 | *SYNJ2* | T | C | 0.513 | 0.008 | 0.001 | 1.11E-12 | 88.570 |
| rs12660376 | *RP1-151F17.2* | C | T | 0.014 | -0.027 | 0.005 | 4.05E-08 | 30.128 |
| rs9296413 | *CRIP3* | T | C | 0.611 | 0.009 | 0.001 | 1.10E-14 | 94.002 |
| rs11238325 | *GRB10* | T | C | 0.734 | 0.007 | 0.001 | 4.50E-08 | 52.594 |
| rs4732339 | *TMEM213* | A | G | 0.585 | 0.006 | 0.001 | 4.05E-08 | 66.092 |
| rs13277721 | *AGO2* | A | G | 0.512 | 0.006 | 0.001 | 1.12E-08 | 70.884 |
| rs10901863 | *CTBP2* | T | C | 0.268 | 0.010 | 0.001 | 2.99E-16 | 86.789 |
| rs67307131 | *PHLDB1* | C | T | 0.347 | 0.008 | 0.001 | 9.98E-13 | 81.058 |
| rs55635402 | *TUB* | G | A | 0.196 | -0.009 | 0.001 | 3.02E-10 | 52.181 |
| rs1126809 | *TYR* | A | G | 0.305 | 0.010 | 0.001 | 6.27E-16 | 75.924 |
| rs1566129 | *NID2* | C | T | 0.586 | -0.006 | 0.001 | 7.44E-09 | 67.779 |
| rs78417468 | *MMP2* | A | G | 0.225 | -0.008 | 0.001 | 1.22E-09 | 54.117 |
| rs72930982 | *CCDC68* | G | A | 0.215 | 0.007 | 0.001 | 3.49E-08 | 47.710 |
| rs11881070 | *TMPRSS9* | T | C | 0.289 | -0.008 | 0.001 | 3.67E-10 | 61.891 |
| rs5756799 | *TRIOBP* | T | G | 0.460 | 0.007 | 0.001 | 2.80E-10 | 74.523 |
| rs36062310 | *KLHDC7B* | A | G | 0.043 | 0.026 | 0.003 | 8.50E-23 | 19.063 |

SNP: single nucleotide polymorphism; MAF: minor allele frequency; SE: standard error.
