# Supplementary material for: Frailty and hearing loss: From association to causation
Source: Front Aging Neurosci. 2022 Sep 7;14:953815. doi: 10.3389/fnagi.2022.953815 (PMC9490320; doi:10.3389/fnagi.2022.953815)
Supplement: Supplementary file 4 [file Table_4.DOCX]

**Supplementary Table 4**. The risk of hearing loss in people with frailty compared to those without frailty.

|  |  | Self-reported HL | | Speech frequency HL | | High frequency HL | |
| --- | --- | --- | --- | --- | --- | --- | --- |
| Model |  | OR (95% CI) | P | OR (95% CI) | P | OR (95% CI) | P |
| sex |  |  |  |  |  |  |  |
|  | Male(n=3605) | 2.604(2.036,3.333) | <0.001 | 1.545(1.203,1.985) | 0.001 | 1.442(1.106,1.884) | 0.007 |
|  | Female(n=3910) | 3.343(2.578,4.350) | <0.001 | 1.266(0.963,1.666) | 0.091 | 1.224(0.976,1.535) | 0.080 |
| age |  |  |  |  |  |  |  |
|  | 40~50(n=2891) | 2.765(2.000,3.822) | <0.001 | 1.650(1.098,2.468) | 0.015 | 1.464(1.083,1.975) | 0.013 |
|  | 50~60(n=2487) | 2.666(1.972,3.614) | <0.001 | 1.290(0.957,1.737) | 0.094 | 1.162(0.885,1.526) | 0.279 |
|  | >60(n=2137) | 3.137(2.325,4.246) | <0.001 | 1.344(1.012,1.786) | 0.041 | 1.463(1.063,2.019) | 0.020 |

Note. Models were adjusted for age, demographic factors (race, education, and poverty ratio, marital, and military), and other risk factors (smoking status, body mass index, noisework, dietary inflammation index, hypertension, diabetes, cardiovascular disease, and chronic obstructive pulmonary disease). HL = hearing loss; OR= odds ratio CI = confidence interval.
