# Supplementary material for: Frailty and hearing loss: From association to causation
Source: Front Aging Neurosci. 2022 Sep 7;14:953815. doi: 10.3389/fnagi.2022.953815 (PMC9490320; doi:10.3389/fnagi.2022.953815)
Supplement: Supplementary file 5 [file Table_5.DOCX]

**Supplementary Table 5.**Linear regression of FI and speech- and high-frequency pure tone average

| Variable | Univariate model | | Multivariate model | |
| --- | --- | --- | --- | --- |
|  | Coefficient (95% CI) | p value | Coefficient (95% CI) | p value |
| Speech-frequency pure tone average | 15.637(11.854,19.420) | <0.001 | 6.438(2.650,10.227) | 0.002 |
| High-frequency pure tone average | 17.818(12.717,22.919) | <0.001 | 9.063(3.801,14.326) | 0.001 |

FI= frailty index.

Multivariate model= regression model adjusted for age, gender, race, education, and poverty ratio, marital, military, smoking status, body mass index, noisework, dietary inflammation index, hypertension, diabetes, cardiovascular disease, and chronic obstructive pulmonary disease.
