# Supplementary material for: Frailty and hearing loss: From association to causation
Source: Front Aging Neurosci. 2022 Sep 7;14:953815. doi: 10.3389/fnagi.2022.953815 (PMC9490320; doi:10.3389/fnagi.2022.953815)
Supplement: Supplementary file 6 [file Table_6.DOCX]

**Supplementary Table 6** Mendelian randomization estimates for the association between frailty index (FI) and hearing loss (HL).

| **outcomes** | **IVW** | | | **MR_Egger** | | | | | **MR_PRESSO** |
| --- | --- | --- | --- | --- | --- | --- | --- | --- | --- |
|  | Cochran Q statistics (df) | I^2^ | P | Intercept (se) | P | Cochran Q statistics (df) | I^2^ | P | P |
| HL | 33.650 (13) | 0.647 | 0.0004 | 0.002 (0.002) | 0.312 | 33.650 (12) | 0.614 | 0.0007 | 0.003 |
| HL ^a^ | 5.102(10) | -1.548 | 0.884 | 0.003 (0.001) | 0.855 | 5.067 (9) | -1.567 | 0.828 | 0.922 |
| FI | 38.715(20) | 0.664 | 0.007 | 0.001 (0.002) | 0.669 | 38.715 (19) | 0.661 | 0.005 | 0.006 |
| FI ^b^ | 12.013 (16) | -0.082 | 0.743 | 0.0001 (0.001) | 0.971 | 12.014 (15) | -0.082 | 0.678 | 0.771 |

IVW= inverse variance weighting;

a=after removing the three outlier SNPs (rs1363103, rs17612102, rs82334).

b= after removing the four outlier SNPs (rs34656207, rs741475, rs10901863, rs1126809, rs1566129, rs9296413).
