# Supplementary figures and images for: Frailty and hearing loss: From association to causation
Source: Front Aging Neurosci. 2022 Sep 7;14:953815. doi: 10.3389/fnagi.2022.953815 (PMC9490320; doi:10.3389/fnagi.2022.953815)

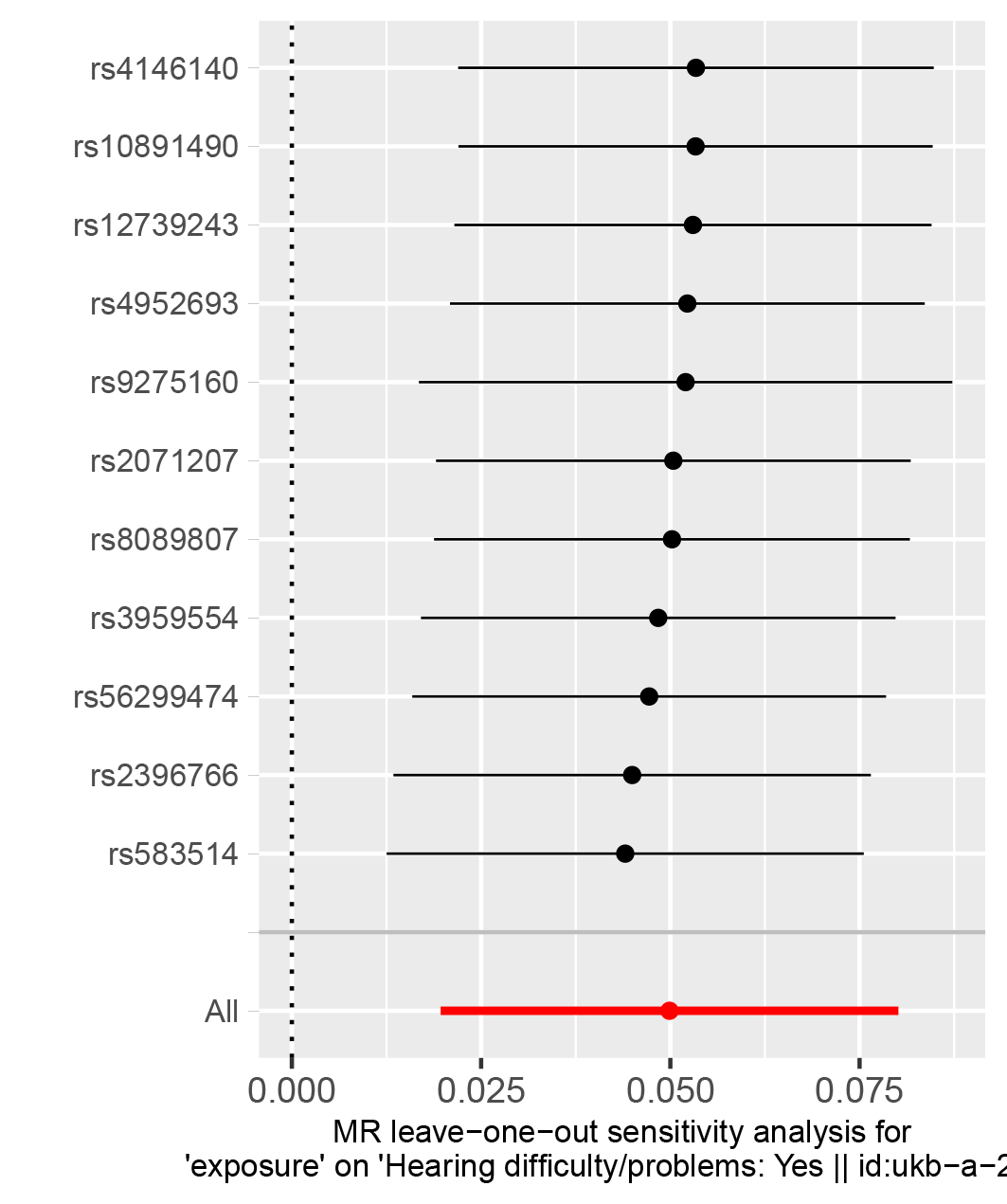

Supplement: Supplementary Figure 1 — Sensitivity analysis of the association between genetically predicted frailty index (FI) and risk of hearing loss (HL). (A) Leave-one-out analysis of the effect of genetically predicted FI and HL; (B) forest plot of the effect of genetically predicted FI and HL; (C) scatter plot of the effect of genetically predicted FI and HL; (D) funnel plot of the effect of genetically predicted FI and HL. [file Image_1.TIF]

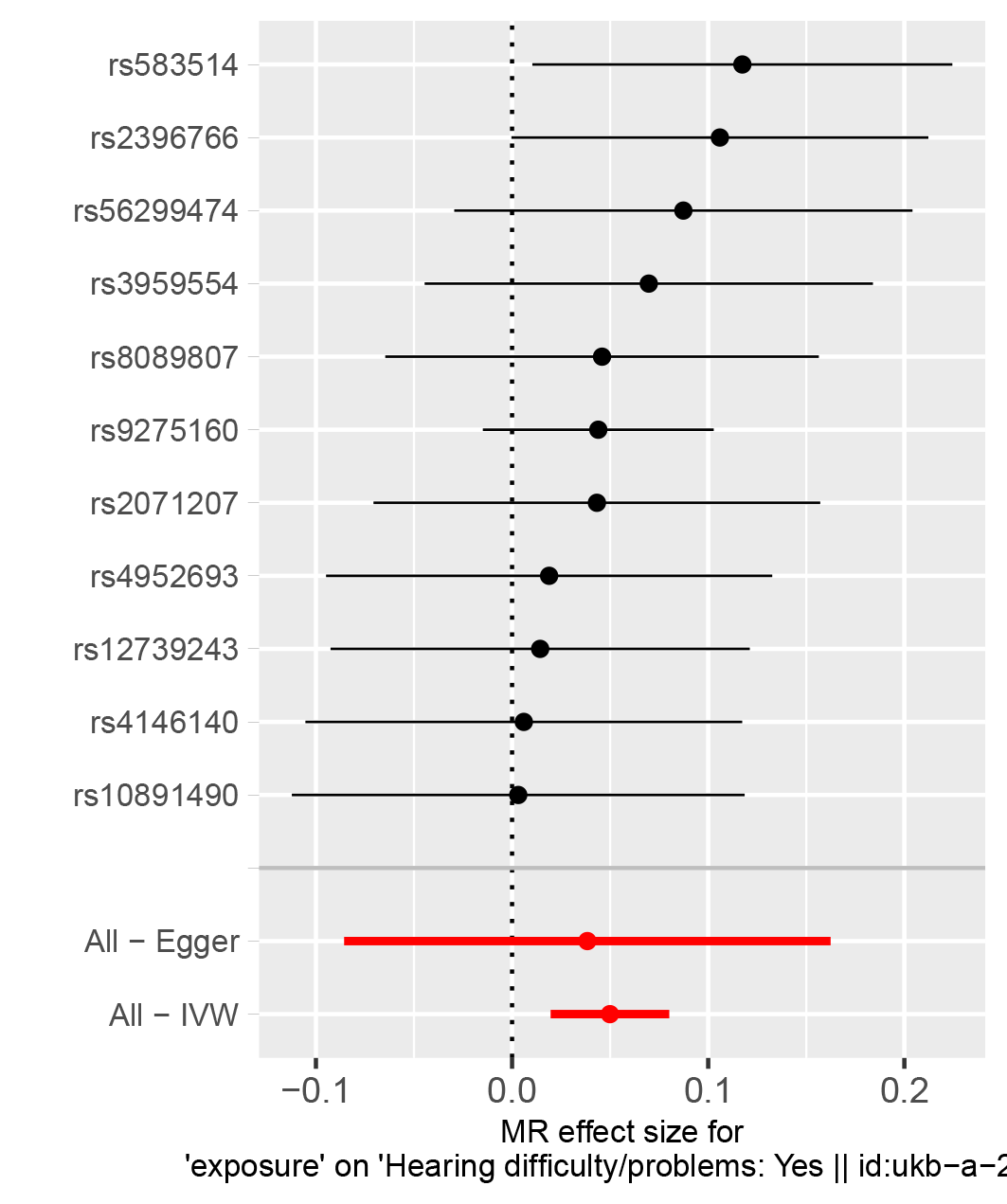

Supplement: Supplementary Figure 2 — Sensitivity analysis of the association between genetically predicted hearing loss (HL) and risk of frailty index (FI). (A) Leave-one-out analysis of the effect of genetically predicted HL and FI; (B) forest plot of the effect of genetically predicted HL and FI; (C) scatter plot of the effect of genetically predicted FI and HL; (D) funnel plot of the effect of genetically predicted FI and HL. [file Image_2.TIF]

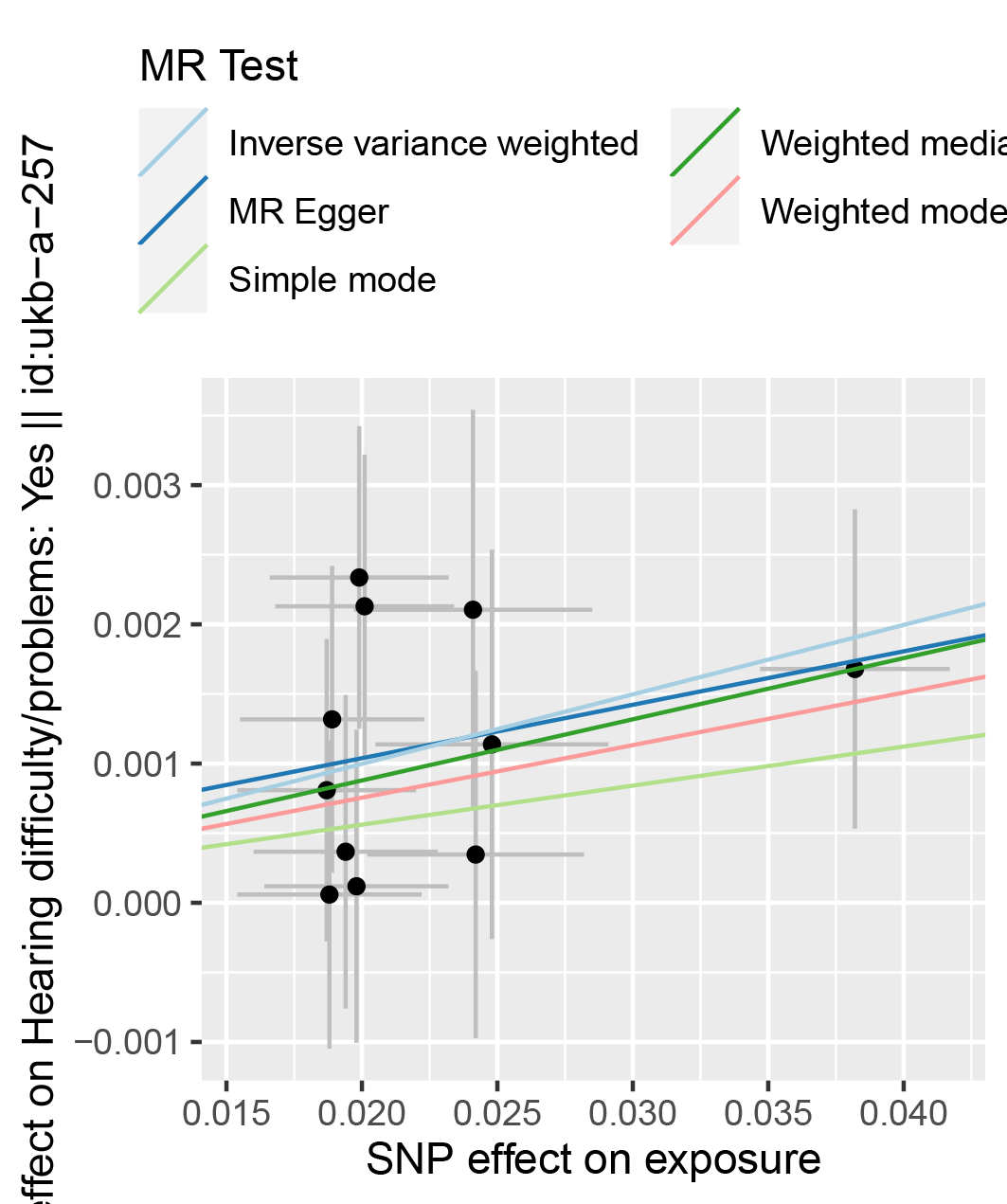

Supplement: Supplementary file 9 [file Image_3.TIF]

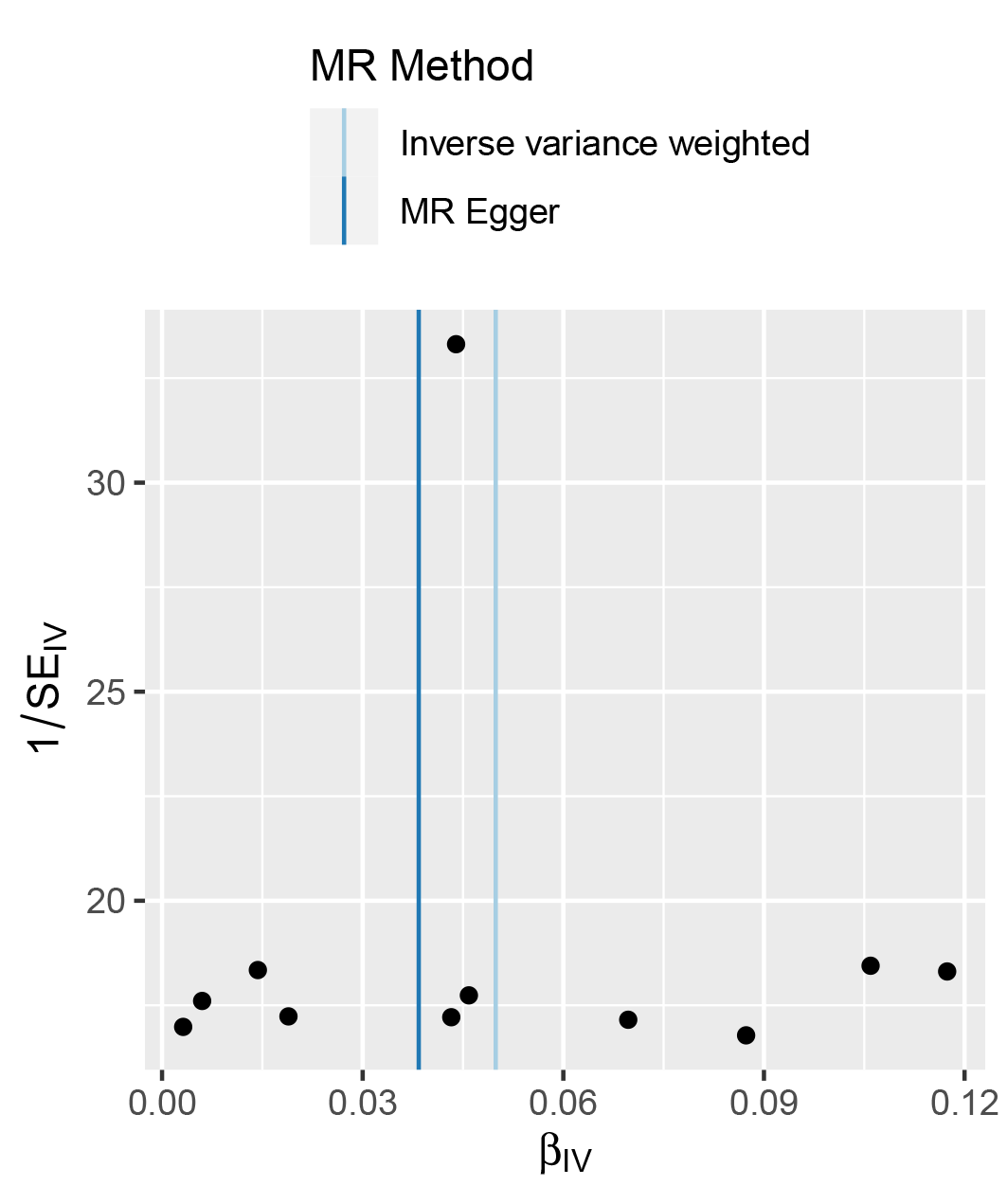

Supplement: Supplementary file 10 [file Image_4.TIF]

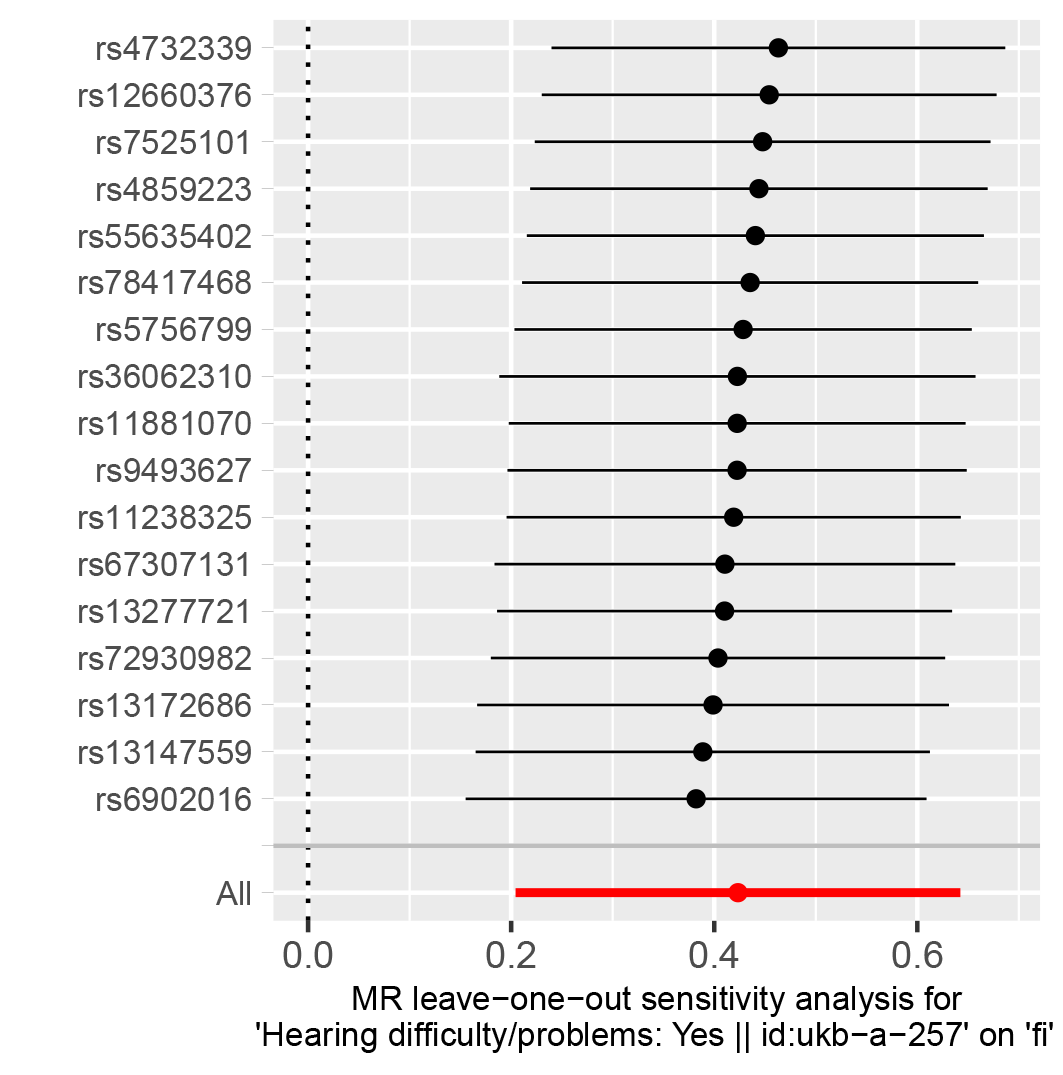

Supplement: Supplementary file 11 [file Image_5.TIF]

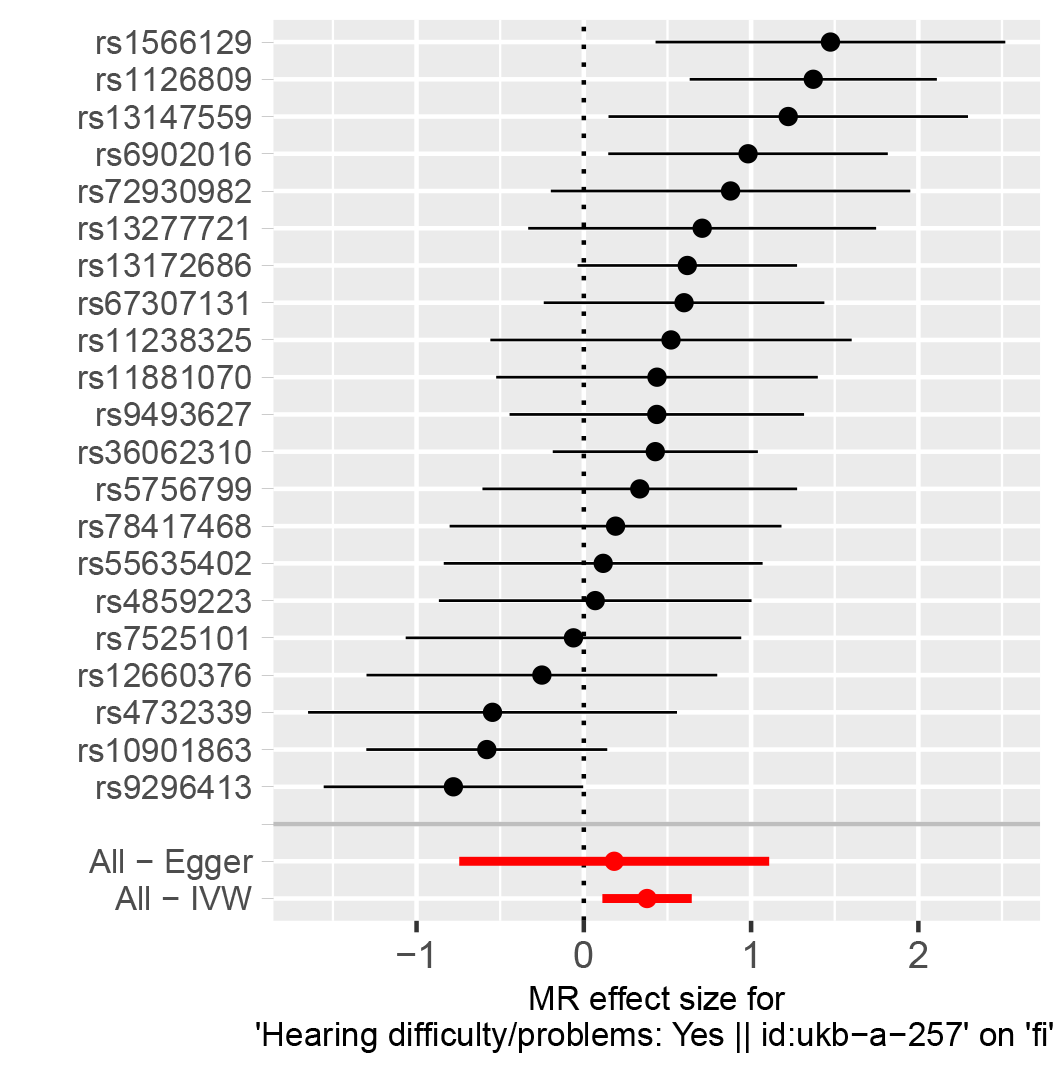

Supplement: Supplementary file 12 [file Image_6.TIF]

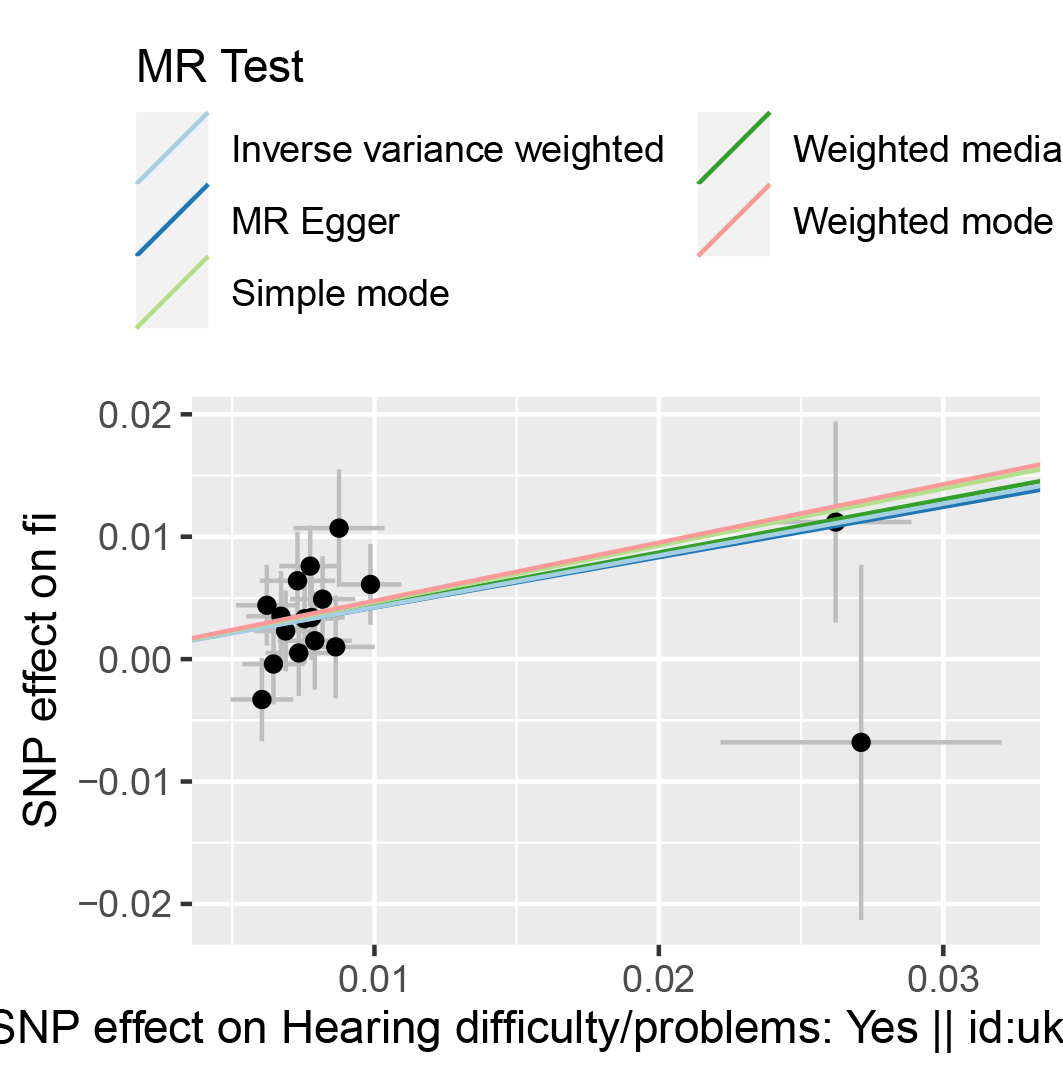

Supplement: Supplementary file 13 [file Image_7.TIF]

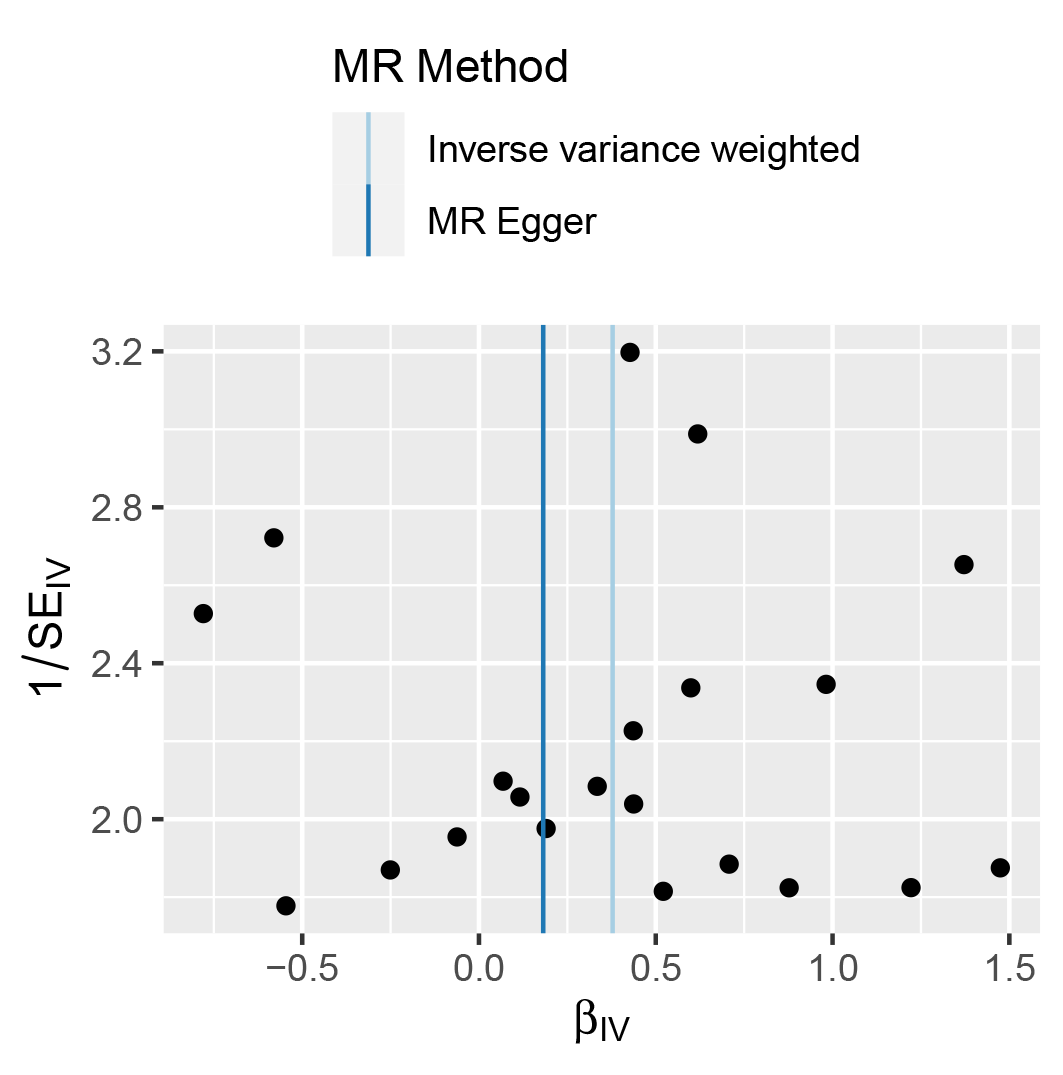

Supplement: Supplementary file 14 [file Image_8.TIF]
